# Current Status of Evidence for a New Diagnosis: Food Addiction-A Literature Review

**DOI:** 10.3389/fpsyt.2021.824936

**Published:** 2022-01-10

**Authors:** Octavian Vasiliu

**Affiliations:** Department of Psychiatry, Dr. Carol Davila University Emergency Central Military Hospital, Bucharest, Romania

**Keywords:** food addiction, behavioral addiction, sugar addiction, eating addiction, obesity

## Abstract

Food addiction is considered an important link for a better understanding of psychiatric and medical problems triggered by dysfunctions of eating behaviors, e. g., obesity, metabolic syndrome, binge eating disorder, or bulimia nervosa. At behavioral level, food addiction has high degrees of similarity with other eating disorders, a phenomenon that creates difficulties in finding specific diagnostic criteria. Food addiction has been also described as “eating addiction” or “eating dependence” by several researchers, who placed the emphasis on the behavior and not on the food itself. High-sodium foods, artificially flavored-foods, rich carbohydrate- and saturated fats-containing foods are triggers for the activation of the same neural pathways, therefore they act similarly to any drug of abuse. Food addiction is considered a disorder based on functional negative consequences, associated distress and potential risks to both psychological well-being and physical health. A clinical scale was validated for the quantification of the eating addiction severity, namely the Yale Food Addiction Severity Scale (YFAS), constructed to match DSM IV criteria for substance dependence. Using this instrument, a high prevalence of food addiction was found in the general population, up to 20% according to a meta-analytic research. The pathogenesis of this entity is still uncertain, but reward dysfunction, impulsivity and emotion dysregulation have been considered basic mechanisms that trigger both eating dysfunctions and addictive behaviors. Genetic factors may be involved in this dependence, as modulators of higher carbohydrate and saturate fat craving. Regarding the existence of potential therapeutic solutions, lorcaserin, antiepileptic drugs, opioid antagonists, antiaddictive agents are recommended for obesity and eating disorders, and they may be intuitively used in food addiction, but clinical trials are necessary to confirm their efficacy. In conclusion, a better understanding of food addiction's clinical profile and pathogenesis may help clinicians in finding prevention- and therapeutic-focused interventions in the near future.

## Introduction

There has been extensive research in the field of behavioral addictions in the last two decades, with an increasingly large number of papers being published about this topic. A simple search in the PubMed database for “behavioral addictions” had found over 64,000 papers published between January 2000 and November 2021. Both the inclusion of the “gambling disorder” together with substance use disorders in the 2013-launched DSM-5 (Diagnostic and Statistical Manual of Mental Disorder, 5th Edition) and the creation of a new diagnosis -“Internet gaming disorder”- mentioned in the section dedicated to “Conditions for further study” have fueled even more the interest for this relatively newly discovered category of addictions (1). Other Internet-related disorders, like social networking, shopping, pornography use, gambling, and binge-watching are actively investigated, and so are the non-Internet related addictions (e.g., video-gaming, television viewing), sport/physical exercise addiction, sex addiction etc. Although they are not currently recognized as independent diagnoses by the American Psychiatric Association or World Health Organization (1–3), people are becoming more and more aware of the negative consequences of their addictions and are looking for help. Data regarding clinical manifestations and risk factors for behavioral addictions are gathering, therefore physicians have to be informed about the vulnerable populations, early signs of addiction, validated methods of detection, and to search for preventive and therapeutic measures (2).

**Figure 1:**
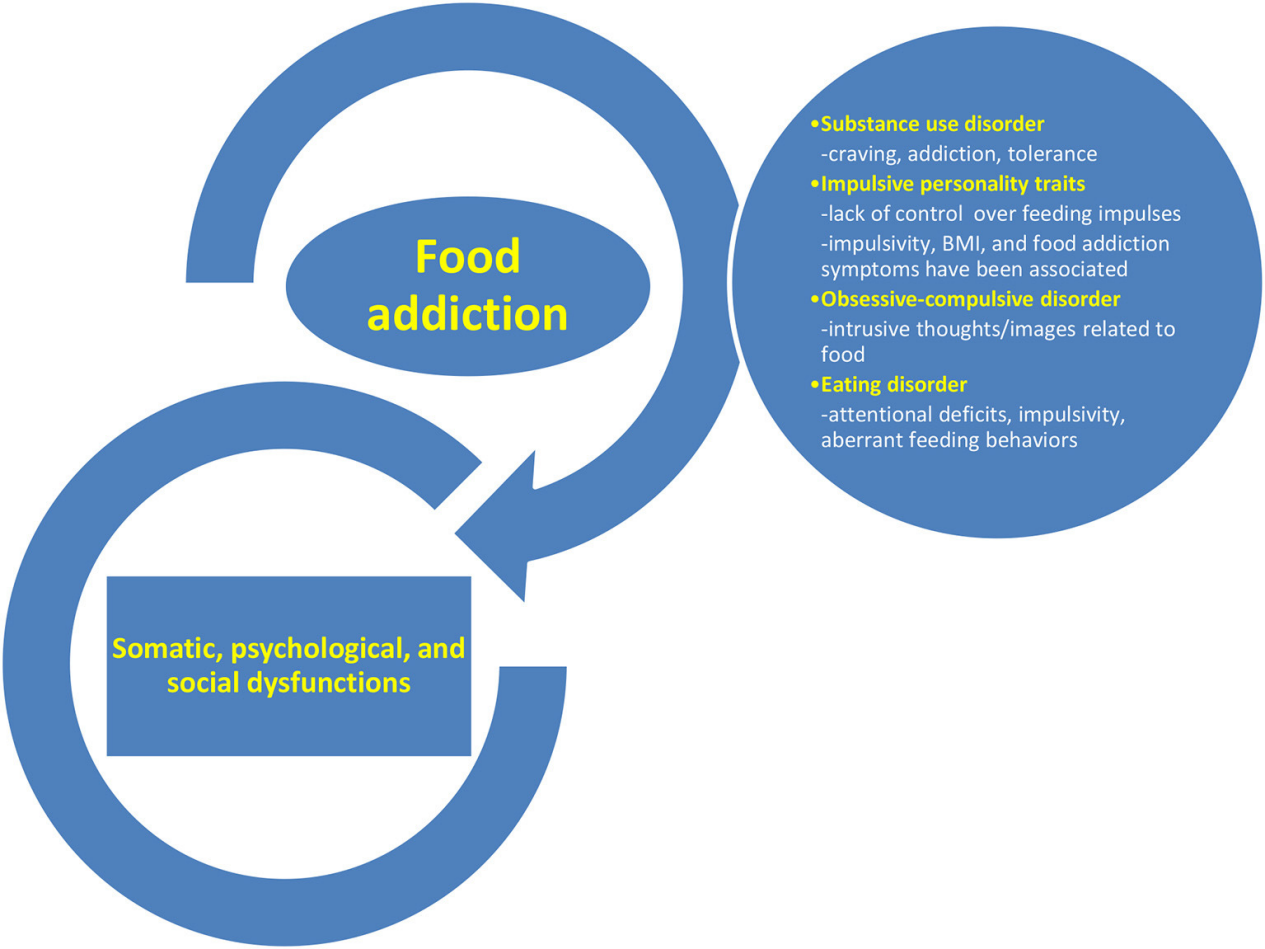
The complex nature of food addiction and its associated health problems.

“Food addiction,” also named “eating addiction,” is one of these recently-cornered behavioral pathologies, but the research of specific diagnostic criteria, measurement methods, prognostic factors, and therapeutic interventions is still in its early phase. Food addiction is a very complex entity because it includes clinical components of an eating disorder (i.e., lack of control over eating behavior) and a substance use disorder (i.e., craving, or continuous use despite awareness of the negative consequences), but also of impulsive personality traits (i.e., dispositional impulsivity is routinely associated with high-risk behaviors including addictive consumption of substances) and an obsessive-compulsive disorder (i.e., intrusive thoughts related to food cues) (Figure 1) (4–6). This addiction may associate various health problems, ranging from psychological/psychiatric (e.g., depressed mood, lower self-esteem based on weight gain, major depressive disorder, binge eating disorder) to somatic (e.g., becoming obese or overweight, development of metabolic imbalances due to selective food consumption, diabetes mellitus, or cardiovascular diseases) or social (e.g., fear of stigmatization due to overweight/obesity or addictive-like behavior) (4, 7–12).

**Figure 2:**
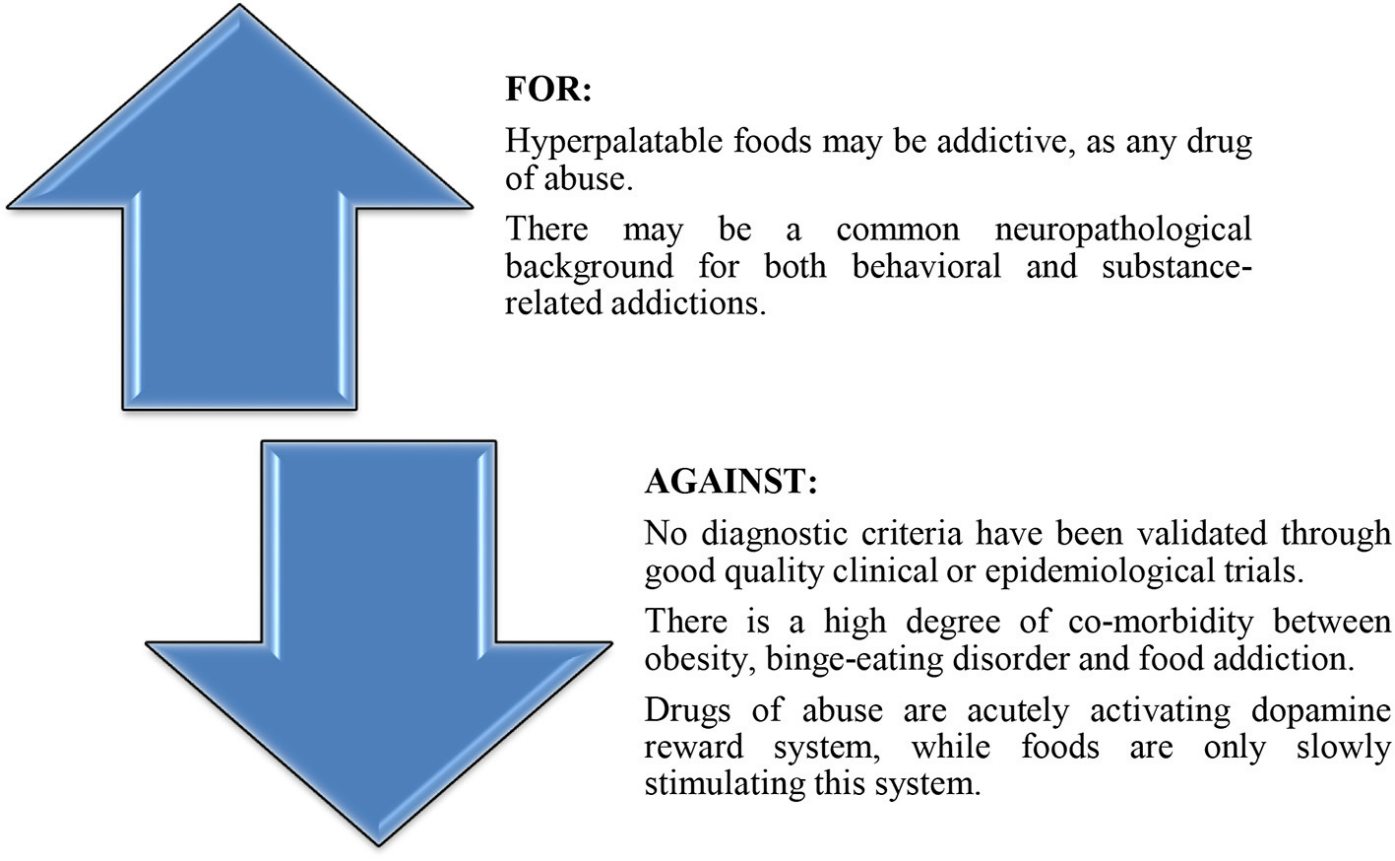
Arguments for and against the diagnosis of “food addiction.”

The construct of “food/eating addiction” is controversial (Figure 2), and several factors tend to negatively interact with its nosographic validation: (a) eating is considered a physiological behavior, therefore distinguishing pathological aspects from whims or culinary preferences is difficult in certain circumstances; (b) the risk to stigmatize socially and culturally-accepted behaviors as being ab-normal is a challenge for mental health specialists, especially if no clear-cut diagnostic criteria exist for this disorder; (c) it is not conceivable to formulate as a therapeutic objective for these patients a complete abstinence, as it is the case with other behavioral addictions; (d) there are no evidence-based therapeutic guidelines, and no clinical or laboratory exams that may be use as definitive, diagnostic methods; (e) there is a high degree of overlap between obesity, binge-eating disorder, bulimia nervosa and food addiction, and separating them solely on clinical basis is difficult (13).

“Eating addiction” is sometimes preferred instead of “food addiction” because both animal and human data are consistent with the existence of addictive eating behaviors, while the evidence for a substance-based food addiction is less consistent (14). Other authors defend the term “food addiction,” stating the substance-based framework is more appropriate than the behavioral-based conceptualization because not all foods are equally addictive, e.g., chocolate vs. high fiber foods, or pizza/fries vs. fruit/vegetables (15). Also, the presence of a behavior (like binging) is not enough to trigger an addictive-like response without the presence of a substance with abuse potential, while the food addiction requires the interaction of certain foods, behavioral patterns of engagement, and individual risk factors for addiction (15). For the purpose of this review we consider that food addiction could be considered a behavioral addiction, since there is no clear evidence that a specific food remains the unique trigger for the abusive behavior in a certain individual, and because eating behavior is still the main focus of research in this field. However, the term “food addiction” is by far the most commonly used term in the literature for this pathology, so it will be used in this review instead of the more adequate term of “eating addiction.”

Food addiction has a high degree of comorbidity with other psychiatric disorders, a phenomenon which is also frequently reported in patients presenting other substance use disorders or behavioral addictions (16, 17). Some authors even state that dual diagnosis is the rule, rather than the exception, especially in clinical samples (8). Possible explanations for this high rate of comorbidity may include self-medication, shared genetic vulnerability, common environment, lifestyle, or neural pathways (16). This clinically- and epidemiologically-supported observation has severe negative consequences reflected in lower treatment adherence, higher risk for physical complications, poorer overall health, poorer self-care, increased suicide or aggression risks, possible legal problems, and greater health burden for patients with dual diagnosis (16). Also, co-addiction is frequently reported in patients, and multiple substance and/or behavioral addictions are being clustered together (17, 18). Mental health professional may, however, be more focused on the acute psychological manifestations of a certain disorder, and ignore or minimize the importance of addictive behaviors, which may occupy the background of the clinical presentation. The use of screening questionnaires or structured interviews may increase the rate of early detection, especially in cases of behavioral addictions, a nosological category that is not yet very well-acknowledged by clinicians (18).

The objective of the current review is to verify if there are enough data in the literature to support the existence of this newly described diagnosis, i.e., “food addiction.” Five dimensions are considered important in order to delineate such a disorder: (1) clinical criteria for diagnosis, (2) one or more validated instruments for the quantification of this disorder's severity, (3) epidemiological data, (4) evidence for specific pathophysiology, and (5) available treatments.

## Methods

A literature review dedicated to finding available evidence for the diagnosis, pathogenesis, epidemiology, methods of structured evaluation, and treatment of food addiction was based upon electronic databases search. The main databases included in the analysis were PubMed, Cochrane, Medscape, Thomson Reuters/Web of Knowledge, APA PsycNet, and the search paradigm was “food addiction” OR “eating addiction” AND “treatment” OR “therapy” OR “epidemiology” OR “diagnosis” OR “clinical criteria” OR “pathogenesis” OR “clinical scales” OR “psychometric instruments.” All papers published between January 1990 and October 2021 were screened for eligibility. Inclusions and exclusions criteria for review are presented in Table 1.

**Table 1:** Inclusion and exclusion criteria.

**Operational criteria**	**Inclusion criteria**	**Exclusion criteria**
Population	All age groups were allowed (children, adolescents and adults) No superior age limit was specified The main diagnoses were food addiction, eating addiction, orthorexia nervosa. Obesity, overweight, metabolic syndrome were accepted only as comorbidities. Other eating disorders (binge eating disorder, bulimia nervosa etc) were allowed as secondary diagnoses Diagnosis should be based on criteria specified by the authors within that paper. No limitation of the diagnostic criteria was included, therefore DSM-based or otherwise fundamented criteria were allowed	Clinical/epidemiological studies that did not specified age limits for their samples The presence of severe somatic or psychiatric co-morbidities with significant impact over cognition, mood, behavior, and overall functionality (e.g., psychotic disorders, refractory bipolar disorder or major depressive disorder, severe neurocognitive disorders)
Intervention	Any type of reviewing method was allowed (systematic, narrative, meta-analytic, mega-analytic, network meta-analytic) Any type of study (clinical/preclinical) was admitted if it corresponded to the pre-defined objective of this review	Studies with unspecified design, population, or statistical methods that have been applied Reviews that did not specified search paradigm, interval for papers collection, criteria for inclusion/exclusion, or those that have used overinclusive search paradigm that did not allow for a distinction between food addiction and other eating behavior dysfunctions
Environment	Both in-patient and out-patient regimen	Unspecified environment
Outcomes	Diagnostic criteria, epidemiology (prevalence, incidence, risk factors), pathophysiology (neurobiological, psychological), and treatment (efficacy, tolerability) of food addiction	All researches that have been using poorly defined outcomes or instruments that have not been validated were excluded. Reviews without clearly pre-defined outcomes were also excluded
Study design	Clinical trials, epidemiological studies, systematic reviews, narrative reviews, meta-analyses. Longitudinal and transversal, retrospective or prospective studies. Animal model studies. Only peer-reviewed papers were allowed	Studies with unspecified or insufficiently defined design Case reports, case series Non-peer-reviewed papers
Language	Any language of publication was admitted if the *in-extenso* published paper was available	

**Figure 3:**
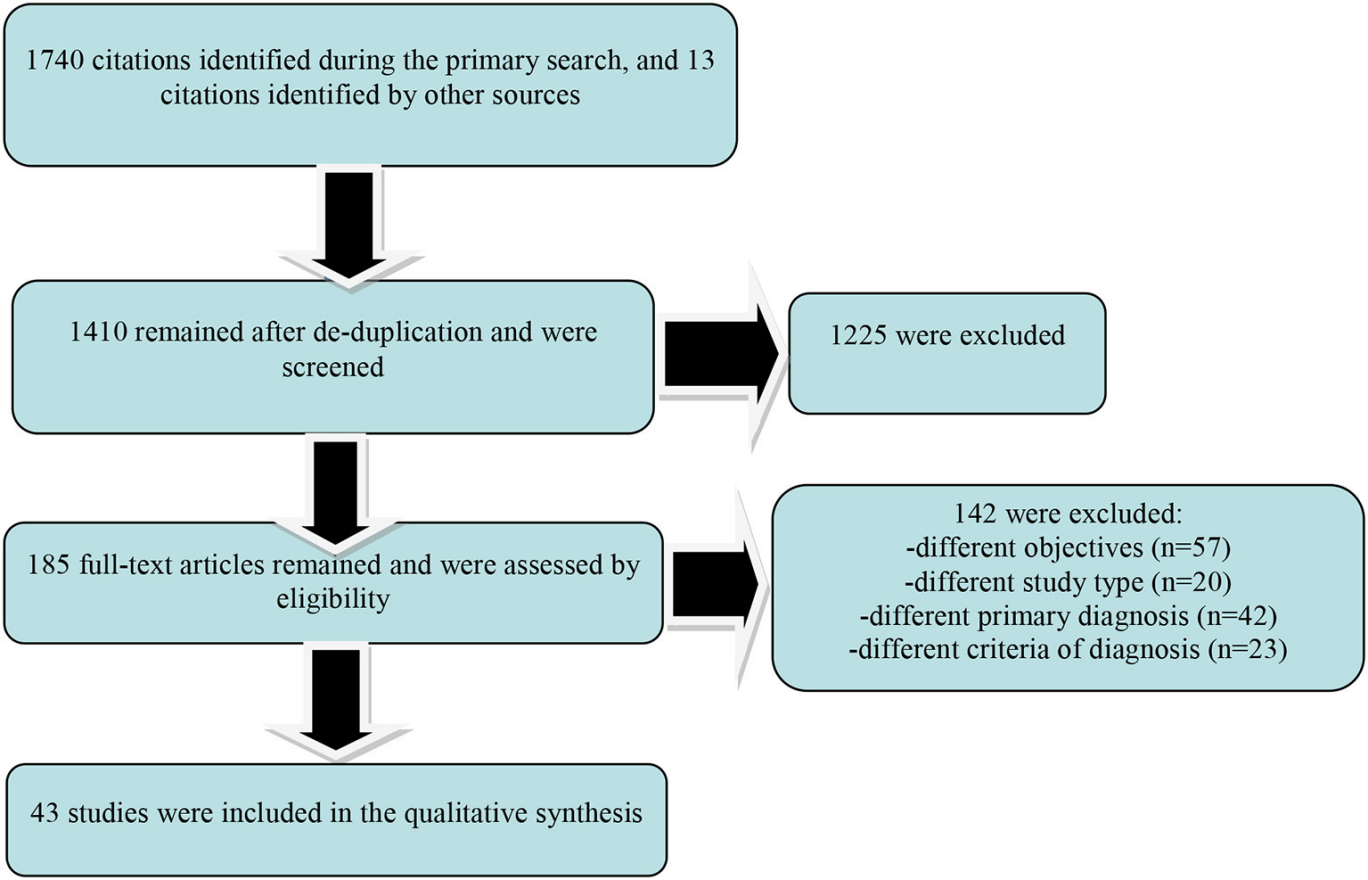
Results of the PRISMA-based search paradigm.

This systematic review is based on the Preferred Reporting Items for Systematic Reviews and Meta-Analyses (PRISMA) statement, and all the data collection, review, reporting, and discussion were conducted according to this statement (Figure 3) (19, 20).

## Results

A number of 1,740 papers surfaced after the primary search, with 13 citations identified by other sources. After filtering these papers using the inclusion/exclusion criteria, only 43 remained for the secondary analysis. An important degree of overlap between papers regarding the information retrieved was detected, because they analyzed in the same time multiple variables of interest for food addiction. A number of 10 papers explored clinical criteria for the diagnosis of food addiction and its subtypes, while 6 papers investigated psychometric properties of a scale dedicated to this pathology. Regarding the pathophysiology of food addiction, 6 papers were retrieved, and 16 papers were reviewed in order to find available information about its epidemiology. Data about the efficacy and/or tolerability of potential treatments for food addiction were extracted from 10 papers, which were mainly reviews.

### Clinical Definitions and Suggested Criteria for Diagnosis

Food addiction is defined as an “eating behavior involving the overconsumption of specific foods in an addiction-like manner” (21). Not all foods are equally addictive, therefore an investigation of the chemical characteristics that may trigger addictive behaviors is needed. Hyperpalatable foods, containing high proportion of saturated fat, sugar, artificial flavors, or sodium have been associated with addictive properties, and public health interventions focused on reducing the impact of addictive drugs may have also a role in targeting obesity and other, related, metabolic diseases (4).

“Sugar addiction” is a subtype of food addiction which is considered to be defined by overconsumption of highly processed foods with rich sugar content (22). Sugar may be addictive through potent reinforcing effects via both gustatory and post-ingestive pathways (23). If sweetness or nutritional signals engage distinctive brain pathways to motivate ingestion is still a matter of debate (23). In mice there are evidence that separate basal ganglia circuitries are responsible for the hedonic and nutritional actions of sugar, and the cell-specific ablation of dopamine-excitable cells in dorsal, but not ventral, striatum inhibited sugar's ability to drive the ingestion of unpalatable foods (23). The non-alimentary stimulation of dopamine-excitable cells in dorsal, but not ventral, striatum determined the ingestion of unpalatable foods (23). In conclusion, sugar recruits a specific dopaminergic circuitry that acts to prioritize energy-seeking over taste quality, and its localization and functioning indicate a possible involvement of the reward system (23). A literature review focused on sugar and food addiction did not find, however, enough evidence to support the existence of sugar addiction in humans, while data from the animal literature suggest that addiction-like behaviors occur only in the context of intermittent access to sugar (as a consequence of limited access to sweet tasting/highly palatable foods, not due to the neurochemical effects of sugar) (22).

“Chocolate addiction” was investigated in a study (*N* = 50 participants, self-defined as “chocoholics”) and the average consumption reported was 60 grams chocolate per week, with specific craving for chocolate about 6 times a week (24). The amount of chocolate consumed was significantly correlated with disinhibition, and 76% of the respondents centered their definition of chocolate addiction on a lack of control around the trigger-food (24). The addictive factor in chocolate was reported to be orosensory, i.e., taste, smell, and texture (16). Consumers who preferred to eat in secret reported a higher degree of aberrant eating (24). Another study (*N* = 15 subjects, self-labeled as “chocolate addicted,” age between 18 and 49) identified several psychological effects reported secondary to the chocolate consumption (an average of 50 g per day of pure cacao): feelings of increased energy, increased concentration ability, and anxiolytic effect during stress (25). Minor withdrawal symptoms were described in 7 cases, and mood disorders, anxiety disorders, pathological personality features were identified in this sample (25).

“Fast food addiction” is yet another controversial subject, with factors like certain nutrients, the characteristics of fast food consumers and the presentation/packaging of fast food being analyzed as potential triggers for dependence (26). High fat and salt content of the fast food, combined with caffeine and rich in sugar beverages served in this type of restaurants contribute to the addictive potential (26). Fast food advertisement provide environmental cues that may trigger addictive overeating, and another important aspect is that obese patients tend to eat more fast food than general population (26). Therefore, obesity may be supported by the rewarding properties of fast food, and lead to tolerance due to leptin and other hormonal imbalance, a phenomenon which further lead to lack of appetite suppression.

“Fat rich food addiction” can be a diagnosable condition, as this food (usually combined with high salt or sugar) is hyperpalatable, and it is liable to be consumed in excess amounts (27). Psychological vulnerabilities like attentional biases have been identified in people presenting tendency toward this type of addiction, while craving and liking for fat has been explored in patients reporting high consumption of saturated fats, meat, butter, sweetened cream desserts, and pastries (27).

Diagnostic criteria for food addiction have mainly been extrapolated from the DSM criteria for substance dependence, based on the model of a common pathogenetic and clinical background for behavioral and drug addictions (28). The consumption of more than initially desired substance/food, or for a longer period of time, intense preoccupation with the substance/food, craving for specific substance/food, and continuous use despite knowledge of adverse events have been the core criteria for the diagnosis of food addiction (7, 28).

### Psychometric Evaluation

The Yale Food Addiction Scale (YFAS) was created in 2009 for the measurement of the food addiction severity, based on the DSM-IV criteria for substance dependence and updated to YFAS 2.0 after the publication of DSM-5 (29, 30). The content of YFAS is represented by 25 questions adapted to assess the full range of diagnostic criteria for dependence in case of overconsuming high fat- or high sugar-containing foods in the last 12 months (29). This scale has good internal consistency, incremental, and convergent validity (29). The YFAS uses two scoring systems for food addiction symptoms (from 0 to 7, according to the DSM-IV diagnosis criteria) and diagnosis (in patients who endorse 3 or more symptoms plus clinical impairment/distress in the past year) (29). There are several limitations in the interpretation of the YFAS scores derived from the fact that it is self-administered and from its restrictive, DSM criteria-based, perspective.

The latest form, YFAS 2.0, is a longer version that contains 35 items, with 4 new criteria added, namely craving, use despite interpersonal or social consequences, failure in role obligations, and use in hazardous situations, according to DSM-5 criteria (30, 31). This version has unifactorial structure and presents high convergent validity with measures of impulsive eating, obesity, and weight cycling (30, 31).

A modified version of the YFAS (mYFAS) was created for administration in large epidemiologic cohorts, by adapting the original scale to 9 items (7 diagnostic criteria plus 2 individual items for clinically significant impairment and distress) (32). The answers to the questions in the symptoms section could be added, resulting in a global 0 to 7 mYscore (32). The internal consistency, the convergent and discriminant validity of the mYscore were adequate and identical to that of the original YFAS (32). The mYFAS and YFAS 2.0 performed similarly on indexes of reliability, convergent validity with related constructs, discriminant validity with distinct measures, and incremental validity supported by associations with frequency of binge eating (33).

A specific variant of YFAS was created for children (YFAS-C), and it has been proven to present adequate internal consistency, convergent validity and incremental validity in predicting body mass index (BMI) (34). This scale has 9 items, scored from 0 (never) to 4 (always) based on the frequency reported in the last 12 months (34). Higher YFAS-C scores correlated not only with higher BMI values, but also with a greater tendency to overeat in response to emotional stimuli (34). YFAS-C scores negatively correlated with satiety responsiveness (although not significantly), which suggests that children with more severe food addiction may be less sensitive to homeostatic indicators related to food consumption (34).

### Prevalence and Risk Factors

Food addiction was found more frequently in patients with obesity, severe depression, higher impulsivity, posttraumatic stress disorder, and attention-deficit hyperactivity disorder in childhood (8). According to a systematic review (*n* = 25 studies, *N* = 196,211 patients, 60% overweight/obese) the prevalence of food addiction was almost 20%, based on the YFAS scores (8). Factors like the age >35, female gender, and higher BMI values were correlated with higher risk for food addiction (8). In obese/overweight sample the incidence of food addiction was double than in the healthy BMI sample (25 vs, 11%), and it was also double in females compared to males (12 vs. 6.4%) (8). In both food addiction and substance use disorders similar clinical, neurobiological, psychopathological and sociocultural risk factors have been found by multiple studies (35).

In two large studies with middle-aged women and older women the prevalence of food addiction measured by mYFAS was reported to vary between 1 and 9%, and it was inversely associated with age and positively correlated with obesity (32). Former smoking status was positively correlated, while physical exercise was negatively associated with food addiction, if age and BMI were controlled for (32).

Eating disorders were comorbid with food addiction in 57.6% of the reviewed cases, comparative to 16.2% in population without diagnoses of eating disorders (8). Patients with bulimia nervosa showed a higher incidence of food addiction compared to people with binge eating disorder in a study (*N* = 815 participants) in which food addiction was also associated with lifetime highest BMI, weight cycling, and other eating pathologies (36). The comorbidity of food addiction and eating disorders in certain patients may reflect a more severe variant of eating pathology (36).

Certain foods, especially processed foods with added sweeteners and fats, demonstrated the highest addictive potential (7). Women with current (*N* = 26) or remitted (*N* = 20) bulimia nervosa were compared to women matched for age and BMI (*N* = 63) in order to evaluate the differences in the YFAS score between the groups (37). All patients with current diagnosis of bulimia nervosa had also criteria for food addiction, according to the YFAS scores, while only 30% of the women with remitted bulimia nervosa had this comorbidity (37). In the same time, none of the participants in the control group received a food addiction diagnosis, therefore it is possible that bulimia nervosa might represent an addiction-like behavior and food addiction might improve when bulimia nervosa symptoms remit (37).

### Pathophysiology

Hyperpalatable foods and drugs of abuse may induce similar behavioral consequences, like craving, continuous use despite negative effects over own health, and reduced control over consumption (4). Reduced D2 receptor availability in obesity and substance use disorder vs. healthy controls may explain a dopamine deficiency in these patients (38). Food addiction, in a similar manner to drugs of abuse, has been supposed to decrease D2 receptors density (39). Individuals who experience less reward to food intake may overeat in order to compensate for this reward dysfunction (40). A systematic review and meta-analysis (*n* = 33 studies) compared patients with A1 allele of the Taq1A polymorphism (associated with a 30–40% lower number of D2 receptors, and being considered a risk factor for drug addiction) and patients without this allele, but no BMI difference between the two groups has been found (41). Although this meta-analysis did not support the presence of a reward deficiency in food addiction, there are reports that individuals with A1 allele are less able to benefit from an intervention aimed to reduce weight, possibly by interfering with increased impulsivity (39). In a trial, greater carbohydrate and fast food craving were associated with A1 vs. A2 allele among Asian Americans college students (*N* = 84), although no BMI differences were found between A1/A1 or A1A2 genotype and A2A2 genotype (41).

A composite index of elevated dopamine signaling (a multilocus genetic profile score) was higher in patients with food addiction diagnosed on the YFAS scoring system, and it correlated positively with binge eating, food cravings, and emotional overeating (42). The relationship between the genetic index of dopamine signaling and food addiction is mediated by certain aspects of reward-responsive overeating (42).

Serotonin has an important role in modulating food and drug reinforcement (43). A ^11^C-DASB-PET study in 60 healthy volunteers reported a negative correlation between cortical and subcortical serotonin transporter (SERT) with BMI values, while tobacco and alcohol consumption did not affect cerebral SERT binding (44). Several anti-obesity drugs act through SERT blockade, which is also an argument for the involvement of serotonergic transmission in the pathogenesis of eating disorders (44).

Foods modulate endogenous opioids and cannabinoids as a function of palatability, and cause delayed increases of dopamine by increasing glucose and insulin (45). The combination of naltrexone and bupropion is marketed for the treatment of obesity, supporting the positive impact of opioidergic neurotransmission in the regulation of food intake, food craving, and other aspects of eating behavior that affect body weight (46).

Dysfunctions of the hypothalamic-pituitary-adrenal axis and CRF have been reported in the withdrawal phase of the addictive cycle (45). Wihdrawal was accompanied by increased CRF expression and CRF1 electrophysiological responsiveness in the central nucleus of the amygdala in rats withdrawn from palatable foods (47).

In a trial with 48 healthy adolescent females, ranging from lean to obese, food addiction scores correlated with significantly greater activation in the anterior cingulate cortex, medial orbitofrontal cortex, and amygdala in response to anticipated food consumption (48). Higher YFAS scores were present in patients presenting greater activation in the dorsolateral prefrontal cortex and caudate in same tests, but less activation in the lateral orbitofrontal cortex, when compared to low scores (48).

In conclusion, similar patterns of neural activation have been found in food addiction and substance use disorders, consisting mainly in elevated activity within the reward circuitry in response to food/drug cues and low activity in the circuitry responsible for inhibition of responses to food intake (48). These data are supported by meta-analyses which evidence greater activation in the amygdala/hippocampus in obese patients compared to normal weight participants in the pre-meal phase, while in the post-meal phase obese individuals had geater activation in the caudate and medial prefrontal cortex vs. normal weight individuals (40). Neural structures involved in the caloric evaluation, arousal, and memory were more active in obese patients before eating, while less activity was found in areas linked to interoceptive processing (40). In the post-meal phase, greater activity was detected in obese patients in areas related to risk vs. reward evaluation and reward processing (40).

A study compared the EEG activity in food-addicted and non-food addicted obese people with alcohol-addicted and non-addicted lean controls (*N* = 20 healthy normal-weight adults, 46 obese participants, and 14 alcohol dependent patients) (49). The results of this study showed the neural brain activity was similar in alcohol addiction and food addiction, a neural pattern consisting of activation in the dorsal and pregenual anterior cingulate cortex, parahippocampal area, and precuneus (49). Another neural pattern was correlated with obesity and consisted of activation in dorsal and pregenual anterior cingulate cortex, posterior cingulate extending into the precuneus/cuneus, and in the parahippocampal and inferior parietal area (49). Food-addicted and non-food-addicted obese people differed by opposite activity in the anterior cingulate gyrus (49).

The involvement of an impaired cognitive control has been suggested in both substance use disorders and behavioral addictions (50). Patients diagnosed with food addiction according to the YFAS scores (*N* = 34) were compared with a control group (*N* = 34) while performing an Eriksen flanker test and an EEG evaluation (50). A higher number of errors in the cognitive test and reduced response-locked components on the EEG (ERN and Pe) have been reported in the food addiction group (50). Therefore, food addiction seems to be associated with impaired performance monitoring, similar to other addictions (50).

A genome-wide association study (GWAS) of food addiction that used mYFAS in 9,314 women of European ancestry showed that two loci met genome-wide significance, and they were mapped to 17q21.31 and 11q13.4 areas (51). These loci could not be related to genes clearly involved in eating behavior (51). The results were significantly enriched for gene members of the MAPK signaling pathway, and no candidate single-nucleotide polymorphism (SNP) or gene for drug addiction was significantly associated with food addiction after correction for multiple testing (51).

Highly processed foods may present similar pharmacokinetic properties with drugs of abuse, i.e., concentrated dose and rapid rate of absorption, due to the addition of fat and/or refined carbohydrates (52). These properties may explain the highly addictive properties of hyperpalatable foods (52). This hypothesis was tested experimentally in a group with 120 participants, who were invited to indicate which foods out of 35 types were most associated with addictive-like eating behaviors (52). Processed food, higher in fat, and glycaemic load were more frequently associated with problematic, addictive-like eating behaviors, probably due to their ability to induce a faster absorption of fat/sugar into the bloodstream (52).

Three main mechanisms have been suggested in the pathogenesis of obesity as an addictive disorder: reward dysfunction, impulsivity and emotion dysregulation (53). The reward dysfunction is based mainly on dopamine neurotransmission abnormalities, and increased activation of the dorsal- and ventral striatum and orbitofrontal cortex by palatable food (53). Impulsivity is another feature shared by obesity and addictive disorders, and it is a reflection of an executive-control deficiency that favors short-term rewards of foods/drugs instead of long-term benefits, and it is correlated with decreased activation of medial prefrontal cortex and other executive-control regions (53). Emotional dysregulation precipitates drugs use or overeating behaviors, and consumption of foods high in fat and/or refined carbohydrates in response to emotional states like stress or negative affect may be relevant for food addiction and obesity (53).

### Treatment

**Figure 4:**
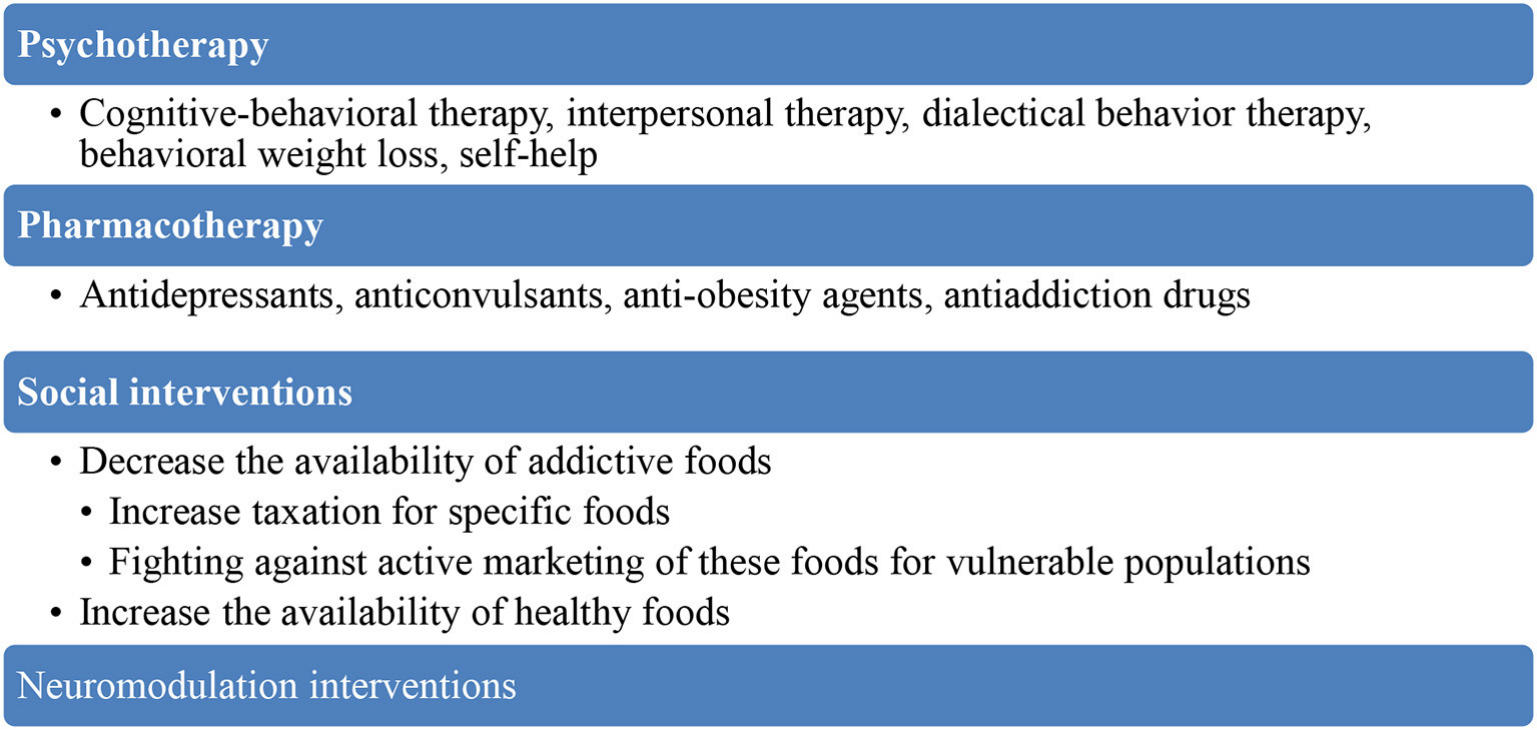
Therapeutic strategies for food addiction.

Food addiction is a complex and multidimensional disorder, with an intricate bio-psycho-social pathogenesis, therefore requiring an integrated therapeutic approach, consisting of psychotherapy, pharmacotherapy, and social oriented support (Figure 4) (35).

Because no well-defined diagnostic criteria for food addiction exist, and the population reported to present this disorder is very heterogeneous, no clinical trial focused on the treatment was identified in the literature. Therefore, data regarding the therapeutic interventions are derived from trials with other, related, eating disorders and from different case management strategies that are based on the presumed neuropathological substrate.

Serotonin, dopamine, and endogenous opioids are considered the main neurotransmitters involved in the dysregulation of eating behaviors, therefore *pharmacologic agents* targeting these systems have been suggested as possible interventions in food addiction. Lorcaserin is a 5HT2C receptor agonist administered for the treatment of obesity, but it has also been recommended for patients diagnosed with drug dependence, obsessive-compulsive disorder, and gambling disorder (43). Selective serotonin reuptake inhibitors were efficient for treatment of binge eating disorder and they were associated with the highest rate of symptoms reduction in placebo-controlled trials (54). Tricyclic antidepressants (desipramine, imipramine) and dual, serotonin and norepinephrine reuptake inhibitors (duloxetine) may also be useful for this pathology (54). Bupropion may also be useful in the treatment of food addiction based on its favorable effects in obese patients with binge-eating disorder (55). Anticonvulsants (topiramate, lamotrigine) have been proven efficient in binge eating disorder, anti-obesity agents may help by targeting consequences of food addiction, while antiaddiction drugs (acamprosate, opioid antagonists) may target the reward system involved in the response to hyperpalatable stimuli (54). The synergistic combination naltrexone/bupropion has been proven more efficient when combined with lifestyle intervention and calorie reduction for patients with obesity than each individual medicine alone (56).

Based on the high degree of overlap between binge eating disorder and food addiction, the main *psychotherapeutic interventions* recommended are cognitive-behavioral therapy (with anti-binging effects maintained on follow-up), interpersonal therapy (decreased binge eating behavior and depressive comorbidity), dialectical behavior therapy (decreased binge eating behavior and associated eating disorder psychopathology), behavioral weight loss, self-help techniques, or combined therapies (54). Overeaters Anonymous (OA) and Weight Watchers International (WW) are self-help groups emphasizing psychological and spiritual components (OA) or behavioral strategies (WW) that provide a framework for developing positive, adaptive, and self-nurturing modalities to cope with eating disorders and obesity (57).

Neuromodulation techniques have also been explored for their potential of reducing craving and addictive behaviors (58). Decrease of substance craving has been demonstrated for transcranial direct current stimulation (tDCS) or repetitive transcranial magnetic stimulation (rTMS) applied to the dorso-lateral prefrontal cortex (DLPFC), an area involved in the inhibitory control, mediated by dopaminergic neurotransmission (58). In adults with food craving (*N* = 19), tDCS improved the percentage of change in cravings rating from pre- to post-stimulation significantly more than sham tDCS (59). *Post-hoc* analyses suggest that active prefrontal tDCS acutely and significantly decreased food cravings for sweet foods more than sham tDCS (59).

*Macro-social interventions* may be focused on changing the availability of addictive foods, on increasing the taxation for these products or for their ingredients, on decreasing the marketing of these type of products for children and adolescents, or on increasing the availability of healthier foods (4).

*Preventive measures* are important for food addiction and for decreasing the incidence of obesity, although it should be noted that not all patients with food addiction are obese or viceversa. Avoidance of triggers for food consumption that may be included in the daily routine (e.g., visual stimuli, like commercials, or olfactory stimuli, like passing by a bakery on the way to work), eating only when someone is feeling hungry (using a 0 to 10 points scale, from starvation to overeating, may be useful in grading the need to eat), improving the emotional control, and regular physical exercise are simple methods that may have a significant impact (60).

## Discussion

Food addiction is a controversial diagnosis which is not included in the current classificatory systems created by either American Psychiatric Association or World Health Organization (1, 3). Also, no unanimously accepted, well-defined diagnosis criteria were detected in the literature during this review. However, the vast majority of the found papers used the same criteria for food addiction that are commonly used for substance use disorders. A set of psychometric instruments has been validated (YFAS, mYFAS, YFAS 2.0, YFAS-C) for quantification of the food addiction severity in adult and children populations.

As in the case of other behavioral addictions, the neurobiological, and psychological factors contributing to the food addiction pathophysiology are common with other substance use disorders. The main explanation for the pathogenesis of food addiction remains a dysfunction in the reward system. Similar clinical, neurobiological, psychopathological, and sociocultural risk factors have been identified in food addiction and substance use disorders (35). Data derived from genetic studies are still sparse, but the less functional dopamine 2 receptor allele has been associated with food addiction and substance dependence (41).

No clinical trial focused on the treatment of food addiction has been identified in the literature, therefore no clear therapeutic recommendation could yet be formulated. A high degree of overlap between food addiction, eating disorders recognized by current classifications, and obesity could be a significant obstacle for designing such trials. The importance of finding a correct conceptual framework for food addiction derives from the same, high degree of overlap between this pathology and obesity. Also, integration of food addiction in the therapeutic management of obese patients could be useful in reaching better outcomes for this population.

This review has inherent limitations based on the scarcity of data derived from clinical trials, which seriously limits the possibility of treatment recommendations. Diagnostic criteria for “food addiction” are controversial, and the heterogeneity of the studied population also limits the possibility of formulating screening strategies that are already implemented for other addictive disorders.

## Author Contributions

The author confirms being the sole contributor of this work and has approved it for publication.

## Conflict of Interest

The author declares that the research was conducted in the absence of any commercial or financial relationships that could be construed as a potential conflict of interest.

## Publisher's Note

All claims expressed in this article are solely those of the authors and do not necessarily represent those of their affiliated organizations, or those of the publisher, the editors and the reviewers. Any product that may be evaluated in this article, or claim that may be made by its manufacturer, is not guaranteed or endorsed by the publisher.
